# Letter to the Editor Regarding ‘Overall Survival with Apalutamide Versus Enzalutamide in Metastatic Castration-Sensitive Prostate Cancer’

**DOI:** 10.1007/s12325-025-03434-x

**Published:** 2025-11-28

**Authors:** Martin W. Schoen, Tito Fojo

**Affiliations:** 1 Medicine Service, Saint Louis Veterans Affairs Medical Center, Saint Louis, MO USA; 2 Department of Medicine, Saint Louis University School of Medicine, Saint Louis, MO USA; 3 Department of Medicine, Columbia University Herbert Irving Comprehensive Cancer Center, New York, NY USA; 4 James J. Peters Bronx Veterans Affairs Medical Center, Bronx, NY USA

**Keywords:** Apalutamide, Enzalutamide, Metastatic prostate cancer, Hormone-sensitive, Castration-sensitive, Comparative efficacy, Observational research

Dear Editor,

We read with interest the study of Bilen et al. [1] and were surprised by the results given that apalutamide and enzalutamide had nearly identical hazard ratios of 0.65 in the TITAN [2] and 0.66 in the ARCHES trials [3]. Thus, the finding of differences in survival was surprising. Since the two drugs have different FDA indications and only enzalutamide is approved for metastatic castration-resistant prostate cancer (mCRPC), it is possible that many patients had enzalutamide prescribed for mCRPC, not metastatic hormone-sensitive prostate cancer (mHSPC), as intended. This concern is heightened by several observations, including: (1) a mean treatment initiation time with enzalutamide almost 2.9 months later than for apalutamide (12.1 vs. 9.2 months), (2) the highest quartile of patients had double the duration of time to initiation of treatment with enzalutamide after diagnosis compared to apalutamide (14.5 vs. 7.2 months), and (3) the larger percentage with prior use of a first generation ARPI and lower use of concurrent ADT with the index ARPI in the non-weighted enzalutamide compared to the apalutamide cohort.

These large differences between the baseline apalutamide and enzalutamide cohorts indicate fundamental differences in patient characteristics. The fact that more than a quarter of patients were started on enzalutamide more than 12 months after diagnosis of metastasis suggests many of those patients had mCRPC. Initiation of an ARPI more than a year after diagnosis of metastasis is typically done for a cause, such as inadequate response to androgen deprivation therapy (ADT) or development of castration resistance. Furthermore, reporting of castration resistance is poor in observational data, and without more comprehensive assessment with imaging reports, PSA, and clinical notes, identification of mCRPC is difficult.

Observational research can inform treatment decisions by comparing therapies, especially when there are no head-to-head trials. To reduce bias and give meaningful results, optimal causal inference methodologies should be used, and inclusion criteria should simulate of a ‘target trial’ [4]. In both the TITAN trial of apalutamide and ARCHES trial of enzalutamide, patients had to be treated within 3 months of ADT or 6 months of ADT if given docetaxel. These time limitations exist to ensure that patients are enrolled with mHSPC since mCRPC can develop within 6–12 months of ADT [5]. To avoid the inclusion of patients with mCRPC, many observational studies adopt intervals such as 120 days from ADT [6, 7] as the limit of time from initial treatment.

To prevent including patients with mCRPC, we suggest an analysis of patients who were treated within 4 months of either metastatic diagnosis or ADT initiation to emulate the target trials and provide a more reliable result. The IPTW method is not adequate if patients have different disease states, and patients with longer times to treatment should be excluded. While this may reduce sample size and statistical power, using optimal methods avoids bias from different FDA indications and more accurately reflects treatment of mHSPC. Without the use of this more rigorous methodology, the results of Bilen et al. should be interpreted with caution.

## Data Availability

Not applicable.
